# Molecular diagnosis of distal renal tubular acidosis in Tunisian patients: proposed algorithm for Northern Africa populations for the *ATP6V1B1*, *ATP6V0A4* and *SCL4A1* genes

**DOI:** 10.1186/1471-2350-14-119

**Published:** 2013-11-20

**Authors:** Donia Elhayek, Gustavo Perez de Nanclares, Slaheddine Chouchane, Saber Hamami, Adnène Mlika, Monia Troudi, Nadia Leban, Wafa Ben Romdane, Mohamed Neji Gueddiche, Féthi El Amri, Samir Mrabet, Jemni Ben Chibani, Luis Castaño, Amel Haj Khelil, Gema Ariceta

**Affiliations:** 1 Laboratory of Biochemistry and Molecular Biology, Faculty of Pharmacy, Monastir, Tunisia; 2 Research Unit, Ciberer, Hospital Universitario Cruces, UPV-EHU, BioCruces, Bizkaia, Spain; 3 Department of Pediatrics, Hospital Fattouma Bourguiba, Monastir, Tunisia; 4 Department of Pediatrics, Hospital Ibn El Jazar, Kairouan, Tunisia; 5 Department of Pediatrics, Hospital Mohamed Ben Sassi, Gabes, Tunisia; 6 Department of Pediatrics, School of Medicine and Odontology, UPV/EHU, Bizkaia, Spain; 7 Division of Pediatric Nephrology, Hospital Universitario Cruces, BioCruces, Bizkaia, Spain; 8 Servicio de Nefrología Pediátrica y Hemodiálisis, Hospital Universitario Materno-Infantil Vall d’Hebron, Passeig de la Vall d’Hebron 119-129, Barcelona 08035, Spain

**Keywords:** Distal renal tubular acidosis, *ATP6V1B1*, *ATP6V0A4*, Tunisian population

## Abstract

**Background:**

Primary distal renal tubular acidosis (dRTA) caused by mutations in the genes that codify for the H + −ATPase pump subunits is a heterogeneous disease with a poor phenotype-genotype correlation. Up to now, large cohorts of dRTA Tunisian patients have not been analyzed, and molecular defects may differ from those described in other ethnicities. We aim to identify molecular defects present in the *ATP6V1B1*, *ATP6V0A4* and *SLC4A1* genes in a Tunisian cohort, according to the following algorithm: first, *ATP6V1B1* gene analysis in dRTA patients with sensorineural hearing loss (SNHL) or unknown hearing status. Afterwards, *ATP6V0A4* gene study in dRTA patients with normal hearing, and in those without any structural mutation in the *ATP6V1B1* gene despite presenting SNHL. Finally, analysis of the *SLC4A1* gene in those patients with a negative result for the previous studies.

**Methods:**

25 children (19 boys) with dRTA from 20 families of Tunisian origin were studied. DNAs were extracted by the standard phenol/chloroform method. Molecular analysis was performed by PCR amplification and direct sequencing.

**Results:**

In the index cases, *ATP6V1B1* gene screening resulted in a mutation detection rate of 81.25%, which increased up to 95% after *ATP6V0A4* gene analysis. Three *ATP6V1B1* mutations were observed: one frameshift mutation (c.1155dupC; p.Ile386fs), in exon 12; a G to C single nucleotide substitution, on the acceptor splicing site (c.175-1G > C; p.?) in intron 2, and one novel missense mutation (c.1102G > A; p.Glu368Lys), in exon 11. We also report four mutations in the *ATP6V0A4* gene: one single nucleotide deletion in exon 13 (c.1221delG; p.Met408Cysfs*10); the nonsense c.16C > T; p.Arg6*, in exon 3; and the missense changes c.1739 T > C; p.Met580Thr, in exon 17 and c.2035G > T; p.Asp679Tyr, in exon 19.

**Conclusion:**

Molecular diagnosis of *ATP6V1B1* and *ATP6V0A4* genes was performed in a large Tunisian cohort with dRTA. We identified three different *ATP6V1B1* and four different *ATP6V0A4* mutations in 25 Tunisian children. One of them, c.1102G > A; p.Glu368Lys in the *ATP6V1B1* gene, had not previously been described. Among deaf since childhood patients, 75% had the *ATP6V1B1* gene c.1155dupC mutation in homozygosis. Based on the results, we propose a new diagnostic strategy to facilitate the genetic testing in North Africans with dRTA and SNHL.

## Background

Distal renal tubular acidosis (dRTA) is defined by the inability of the kidney to acidify urine, in presence of metabolic acidosis [1,2]. Patients present with hyperchloraemic metabolic acidosis, hypokalaemia [3], bone disease and, commonly, nephrocalcinosis. Some affected individuals also exhibit hearing impairment [3,4]. Malfunction of the H + −adenosine triphosphatase (ATPase) pumps, or Cl/HCO3- (anion exchanger AE1) at the α-intercalated cells of the distal nephron causes failure to excrete acid or reabsorb bicarbonate, respectively, and leads to primary dRTA [1,5].

Primary dRTA caused by mutations of H + −ATPase pump subunits is wide genetic and clinically heterogeneous [6]. In 1999, Karet *et al* demonstrated that, in families with classic dRTA and early sensorineural hearing loss (SNHL), which became clinically evident from birth to late childhood, the disease was caused by mutations in the *ATP6V1B1* gene, encoding the B subunit of the H + ATPase pump [7]. Later, in 2000, Smith *et al* found that some families with dRTA and normal audiometry presented with mutations in the *ATP6V0A4* gene, which encodes for the A4 subunit of the H + ATPase pump [8].

Despite those original molecular descriptions distinguished between *ATP6V1B1* and *ATP6V0A4* gene mutations based on the presence or absence of early SNHL, phenotype overlapping between both subgroups of patients makes that classification inaccurate. Indeed, Stover *et al* demonstrated that young adult patients with *ATP6V0A4* mutations could develop mild SNHL in the long term [9]. Further, some dRTA patients without early SNHL were found to present mutations in the *ATP6V1B1* gene as well [10,11].

Further, autosomal dominant (AD) and autosomal recessive (AR) dRTA have also been associated with mutations in the *SLC4A1* gene encoding the human AE1 [12-15], which also plays a central role in the maintenance of acid–base balance [16,17]. Overall, lack of phenotype-genotype correlation supports the need of genetic analysis in dRTA patients.

Up to now, small series of patients with dRTA from different ethnic backgrounds had been genetically studied [7-9,11,18]. Despite a few data on Tunisians have been reported [8,9,11], to our knowledge large cohorts of Tunisian patients with dRTA have not yet been analyzed. In order to establish the genetic diagnosis of primary dRTA in Tunisia, this study aimed to identify the molecular defects that occur in *ATP6V1B1*, *ATP6V0A4* and *SLC4A1* genes using a proposed algorithm.

## Methods

### Patients

We studied 25 children (19 boys and 6 girls) from 20 unrelated families of Tunisian origin, who had been clinically diagnosed of primary dRTA [19]. The study group was recruited after deep review of every medical record with the diagnosis of dRTA, and recall of those affected subjects in four Tunisian hospitals: Hospital Fattouma Bourguiba of Monastir (13 patients from 10 families), Hospital Ibn al-Jazzar of Kairouan (9 patients from 7 families), Hospital Mohamed Ben Sassi of Gabes (2 patients from 2 families), and Hospital Tahar Sfar of Mahdia (one patient). After the four Medical Ethical Committees belonging to the hospitals from which patients were recruited approved the project, and after informed consent was given, we recorded patients’ data regarding family history, symptoms, acid–base and biochemistry at presentation, as well as kidney ultrasound. Clinical diagnosis of dRTA was based on the simultaneous presence of non-gap metabolic acidosis (plasma bicarbonate <20 mEq/L) and inability to reduce urinary pH below 5.5 [20]. Additional features were dehydration, failure to thrive, polyuria, and hypokalemia as described [19]. Hearing was assessed by pure-tone audiometry and/or auditory evoked responses, and the presence of SNHL was graded according to the “Bureau International d’Audiophonologie” recommendations (http://www.biap.org). Moreover, 17 asymptomatic patients’ parents without metabolic acidosis were also studied.

### DNA analysis

**Figure 1:**
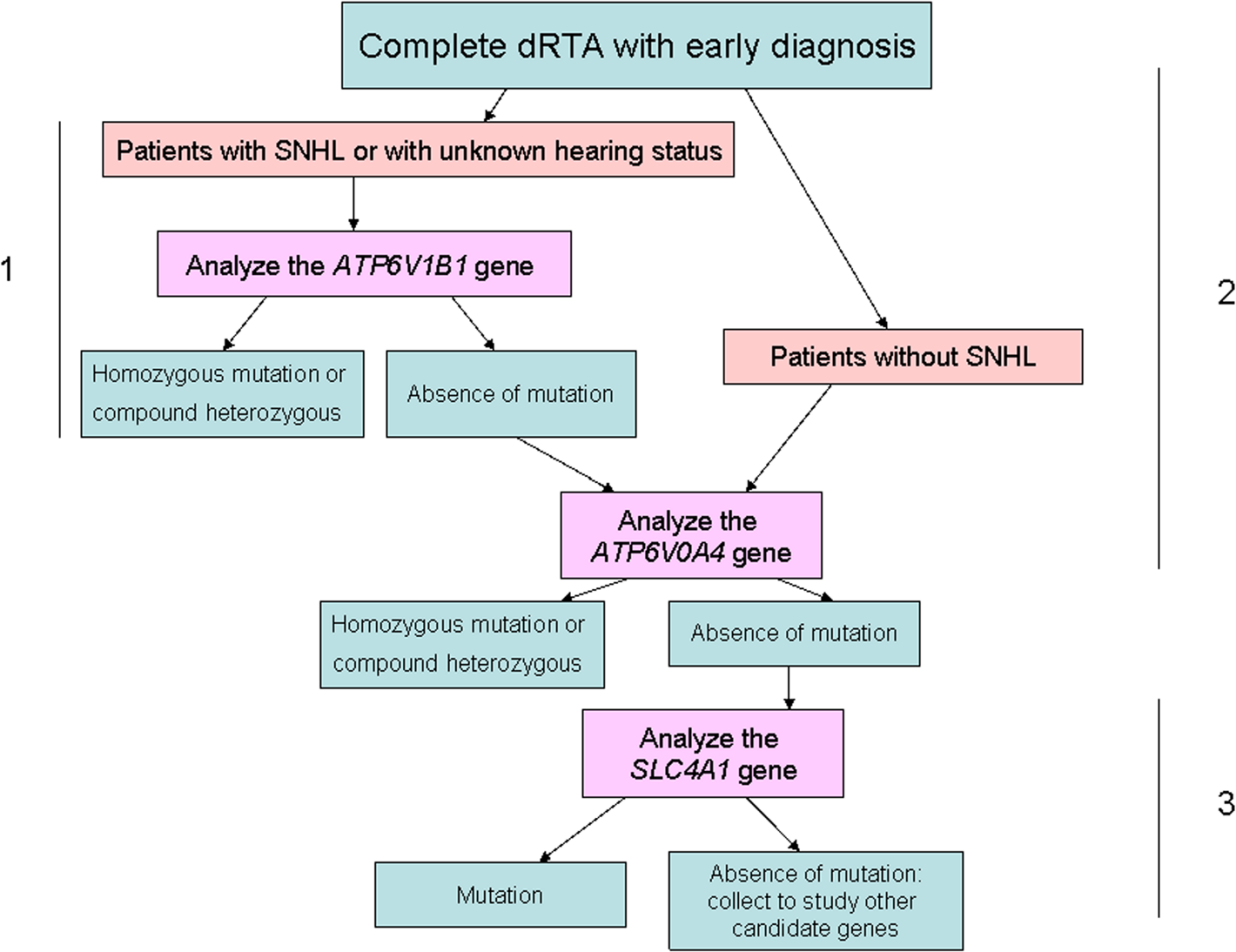
**Algorithm followed for the genetic analysis.** 1, 2 and 3 (the different steps of the analysis).

Peripheral blood samples from the patients were collected on EDTA tubes. DNAs were extracted by the standard method of phenol/chloroform [21]. We planned to optimize the study of the three genes described as being responsible for primary dRTA (*ATP6V1B1*, OMIM *192132; *ATP6V0A4*, OMIM *605239 and *SLC4A1*, OMIM ^+^109270) using the following algorithm: first, we analyzed the *ATP6V1B1* gene for those patients with dRTA and SNHL or unknown hearing status. Afterwards, we studied the *ATP6V0A4* gene in those patients with dRTA but normal hearing, and in those who were negative for *ATP6V1B1* gene mutation screening despite presence of SNHL. Finally, we planned to investigate the *SLC4A1* gene in those remaining patients with dRTA but no identified mutations in either *ATP6V1B1* or *ATP6V0A4* genes (Figure 1).

We used previously reported primers [7] for the polymerase chain reaction (PCR) amplification of the exonic and flanking intronic regions of the *ATP6V1B1* gene. Amplification was conducted for the coding regions and flanking intronic sequences of *ATP6V0A4* using a set of newly designed primers pairs. Structural analysis of the *SLC4A1* gene was performed using both previously described [22] and newly designed primers (for the amplification of the three first exons). Two overlapping regions in *SLC4A1* gene intron 3 (one for the potential kidney promoter and another for the possible 5’ sequence of the transcript expressed in kidney) were also analyzed [22]. In all cases, primer sequences and PCR conditions are available on request. Amplified products were purified using ExoSap (USB, Cleveland, OH, USA) and sequenced in both strands on an ABI3130xl Genetic Analyzer (Applied Biosystems, Foster City, CA, USA). Sequences were analyzed with Sequencing Analysis software v.5.2 (Applied Biosystems) and compared with the references sequences of each gene (Ensembl identifiers: *ATP6V1B1*: ENSG00000116039, *ATP6V0A4*: ENSG00000105929 and *SLC4A1*: ENSG00000004939), using SeqScape software v.2.5 (Applied Biosystems).

### Mutation prediction

The potential pathogenic effect of novel missense mutations was analyzed *in silico* using SIFT (http://sift.jcvi.org/) and Polyphen-2 (http://genetics.bwh.harvard.edu/pph2/) software packages, which are able to predict whether an amino acid substitution may affect protein function, based on sequence homology and physical properties of substituted amino acids.

## Results

### dRTA kindreds

Twenty five Tunisian children (19 males) of 8 (0.6-20) (median, range) years of age were diagnosed with primary dRTA at 6.27 (0.8-36) months of life. At diagnosis, most relevant symptoms were feeding difficulties, dehydration, and failure to thrive (Table 1). One of the patients (case 7.2) was asymptomatic at recruitment; he was diagnosed by molecular familiar screening, as he had an affected brother (case 7.1) and has developed the symptoms later. All patients presented with a non-gap metabolic acidosis (plasma pH 7.23 ± 0.15; bicarbonate 9.67 ± 0.62 mEq/L), and inability to renal acidification, as shown by alkaline urine (urine pH 7 ± 0.67). Further, common findings were hypokalemia (15 cases had K < 3.5 mEq/L), diffuse nephrocalcinosis which was demonstrated in all but three children (nephrocalcinosis was not observed in subjects 7.1 and 19.1 and ultrasound data was not available in case 11.1), rickets (5 cases), and total hearing loss/cophosis (16 cases). Unexpected hyperkalemia was observed in a 1 month old patient (case 13.1), possibly due to extreme acidosis and potential inadequate blood draw (Table 1). The disease was inherited following an AR pattern, and remarkably consanguinity rate was very high in the study group (80%) (4 families in first degree of consanguinity, 5 families in second degree, 3 families in third degree and 4 families reported consanguinity in an unknown status).

**Table 1:** Clinical presentation of the 25 Tunisian children with dRTA

**Case**	**Symptoms**	**Age at diagnosis (months)**	**P pH**	**P Bicarbonate mEq/L**	**P K mEq/L**	**U pH**	**Nephro calcinosis**
1.1	Vomits, growth failure	6	7.3	10.4	2.26	7	Yes
2.1	Vomits, growth failure	7	7.22	9.7	3	6.5	Yes
3.1	Growth failure	1.3	7.14	7	2.3	6	Yes
3.2	Speech delay, growth failure, rickets	36	7.21	12.3	2.7	7	Yes
4.1	Dehydration	1.3	7.21	10.4	4.1	6.5	Yes
5.1	Vomits, diarrhea, growth failure	3	7.3	6.9	4.06	7.5	Yes
5.2	Growth failure, dehydration	3.5	7.25	8.3	4.65	6	Yes
6.1	Growth failure, fever	3	7.34	12.4	3.82	7	Yes
7.1	Loss of weight, dehydration, fever, vomits, diarrhea	4	7.29	12.1	2.43	6	No
7.2	Growth failure, dehydration, polyuria, urine infection	11	7.17	8.5	2.9	7	Yes
8.1	Fever, vomits, growth failure	3.3	7.3	8.6	2.68	7	Yes
9.1	Vomits, dehydration, vitamin D-resistant rickets	1	7	NA	4.22	8	Yes
10.1	Vomits, dehydration, leukocyturia	3	7.3	11.4	2	7	Yes
10.2	Dehydration, vomits	6	7.4	14.5	3.2	6.5	Yes
11.1	Diarrhea, vomits, polyuria, rickets	16	7.32	11.6	3.12	NA	NA
12.1	Vomits, dehydration, growth failure	5	7.28	NA	2.4	7.5	Yes
13.1	Vomits after each feeding	1	7.23	11.3	7.2	7	Yes
14.1	Dehydration, respiratory distress	0.8	7.1	3.4	2	8	Yes
15.1	Dehydration, vomits, feeding difficulties	1.5	7.19	8.4	3.5	7	Yes
16.1	Vomits, feeding difficulties, severe dehydration, diarrhea, vitamin D-resistant rickets, severe osteopenia	18	7.18	9.6	NA	7.5	Yes
16.2	Hypotonia, failure to thrive, severe vitamin D-resistant rickets, inability to walk, dumb	17	7.15	9.4	4.1	NA	Yes
17.1	Vomits, dehydration, respiratory infection, breathing difficulties	0.9	7.29	12.5	3.11	8	Yes
18.1	Dehydration, vomits, polyuria	2	7.15	6.1	3.9	7	Yes
19.1	Bronchiolitis and cough	3	7.13	8.8	3.8	8	No
20.1	Dehydration, axial hypotonia, hypotrophy	2.3	7.39	9	2.2	8	Yes
		6.27 (0.8–36)*	7.23 ± 0.15**	9.67 ± 0.62**	3.3 ± 0.83**	7 ± 0.67**	
*** (mEq/L)			7.35-7.45	22-28	3.5-5	5.5-6.5	

*Abbreviations*: *P* (plasma), *U* (urine), *NA* (not available). *:median (range); **:mean value ± SD; ***:normal ranges.

### Mutation detection

All molecular results are indicated in Table 2. Following the strategy described above (Figure 1), we analyzed the *ATP6V1B1* gene in 16 index cases who presented dRTA and SNHL (12 cases) or an unknown hearing status (4 cases). Mutations were detected in homozygosis in 13 probands. Afterwards, we investigated the *ATP6V0A4* gene in the remaining 3 patients without *ATP6V1B1* mutations, and in those 4 index cases with no hearing loss. Mutations were detected in all 7 probands, 5 of them in homozygosis (4 with normal hearing and 1 with unknown hearing status) and two in heterozygosis, in which loss of hearing was present in one case and the other had an unknown hearing status. Further, in those two patients with isolated heterozygous *ATP6V0A4* gene mutations, we screened for the second mutations in the *SLC4A1* gene, in order to complete the genetic diagnosis of all known causative genes of primary dRTA. We did not find any *SLC4A1* gene mutation, and therefore, the second mutation in those two siblings, if present, remained unidentified.

**Table 2:** Genetic results of the 25 Tunisian patients with dRTA, according to family history and presence of sensorineural hearing loss (SNHL)

**Case**	**Origin**	**Consanguinity**	**SNHL**	**Gene**	**Mutation**	**Protein**
1.1	Monastir	Yes	Yes	*ATP6V1B1*	c.175-1G > C	p.?
2.1	Monastir	Yes	Yes	*ATP6V1B1*	c.1155dupC	p.Ile386Hisfs*56 (p.I386Hfs*56)
3.1	Monastir	Yes	Yes	*ATP6V1B1*	c.1155dupC	p.Ile386Hisfs*56 (p.I386Hfs*56)
3.2	Monastir	Yes	Yes	*ATP6V1B1*	c.1155dupC	p.Ile386Hisfs*56 (p.I386Hfs*56)
4.1	Monastir	Yes	Yes	*ATP6V1B1*	c.1155dupC	p.Ile386Hisfs*56 (p.I386Hfs*56)
5.1	Monastir	Yes	NA	*ATP6V1B1*	c.1102G > A	p.Glu368Lys (p.E368K)
5.2	Monastir	Yes	Yes	*ATP6V1B1*	c.1102G > A	p.Glu368Lys (p.E368K)
6.1	Monastir	No	Yes	*ATP6V1B1*	c.1155dupC	p.Ile386Hisfs*56 (p.I386Hfs*56)
7.1	Monastir	Yes	Yes	*ATP6V1B1*	c.1155dupC	p.Ile386Hisfs*56 (p.I386Hfs*56)
7.2	Monastir	Yes	Yes	*ATP6V1B1*	c.1155dupC	p.Ile386Hisfs*56 (p.I386Hfs*56)
8.1	Monastir	Yes	Yes	*ATP6V1B1*	c.1155dupC	p.Ile386Hisfs*56 (p.I386Hfs*56)
9.1	Kairouan	No	NA	*ATP6V1B1*	c.175-1G > C	p.?
10.1	Kairouan	Yes	Yes	*ATP6V1B1*	c.1155dupC	p.Ile386Hisfs*56 (p.I386Hfs*56)
10.2	Kairouan	Yes	Yes	*ATP6V1B1*	c.1155dupC	p.Ile386Hisfs*56 (p.I386Hfs*56)
11.1	Kairouan	Yes	NA	*ATP6V1B1*	c.175-1G > C	p.?
12.1	Gabes	No	Yes	*ATP6V1B1*	c.1155dupC	p.Ile386Hisfs*56 (p.I386Hfs*56)
13.1	Gabes	Yes	Yes	*ATP6V1B1*	c.1155dupC	p.Ile386Hisfs*56 (p.I386Hfs*56)
14.1	Mahdia	Yes	No	*ATP6V0A4*	c.1221delG	p.Met408Cysfs*10 (p.M408Cfs*10)
15.1	Kairouan	Yes	NA	*ATP6V0A4*	c.16C > T	p.Arg6* (p.R6*)
16.1	Kairouan	Yes	Yes	*ATP6V0A4*	c.2035G > T	p.Asp679Tyr (p.D679Y) heterozygous
16.2	Kairouan	Yes	Yes	*ATP6V0A4*	c.2035G > T	p.Asp679Tyr (p.D679Y) heterozygous
17.1	Kairouan	Yes	No	*ATP6V0A4*	c.16C > T	p.Arg6* (p.R6*)
18.1	Monastir	Yes	No	*ATP6V0A4*	c.16C > T	p.Arg6* (p.R6*)
19.1	Monastir	No	NA	*ATP6V0A4*	c.1739 T > C	p.Met580Thr (p.M580T) heterozygous
20.1	Kairouan	Yes	No	*ATP6V0A4*	c.16C > T	p.Arg6* (p.R6*)

Otherwise stated, mutations are present in homozygosis.

cDNA and protein numbering according to Ensembl identifiers: *ATP6V1B1*: ENSG00000116039, and *ATP6V0A4*: ENSG00000105929.

*Abbreviations*: *SNHL* (sensorineural hearing loss), *NA* (Not available).

**Figure 2:**
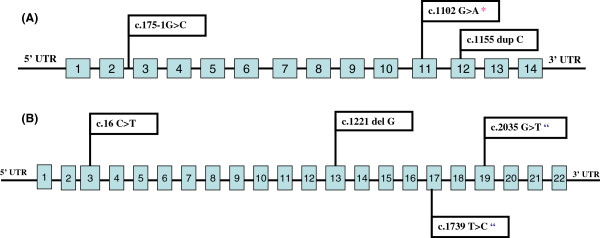
**Schematic representation of the ATP6V1B1 (A) and the ATP6V0A4 (B) genes with the localization of the mutations found in this study.** *: Novel mutation. “Heterozygous.

On the basis of this genetic diagnosis strategy in patients with dRTA, which is based on the presence of SNHL, the initial *ATP6V1B1* gene screening resulted in a mutation detection rate of 81.25% (26/32 alleles). After *ATP6V0A4* gene analysis, the mutation detection rate increased up to 95% of the probands (38/40 alleles). That diagnostic approach was reinforced by the fact that no phenotypic difference, apart from the presence or absence of hearing impairment, was observed between patients with *ATP6V1B1* vs. *ATP6V0A4* mutations in the studied cohort. All the mutations identified in this study were distributed throughout both *ATP6V1B1* and *ATP6V0A4* genes (Figure 2), so no hot-spot was identified in any of the genes.

### Mutations in the *ATP6V1B1* gene

Analysis of the coding regions and flanking intronic sequences of the *ATP6V1B1* gene showed the presence of three different mutations in 13 families (Table 2). One of them was a missense mutation (c.1102G > A; p.Glu368Lys), another was a frameshift mutation (c.1155dupC; p.Ile386fs), and the third was a splice-site mutation (c.175-1G > C; p.?). The first one had not yet been described.

The novel missense mutation in exon 11 (c.1102G > A; p.Glu368Lys) was identified in two siblings (cases 5.1, 5.2) in the current study. That guanine to adenine substitution leads to the change of the amino acid glutamine to lysine. We did not perform a functional analysis of the mutated protein, but a molecular difference between these two residues (glutamic acid is an acidic amino acid, whereas lysine is a basic one) supports the hypothesis that this replacement may affect the structure of the protein subunit, thus the function, folding, or trafficking of that protein. Indeed, glutamic acid in position 368 remains highly conserved among species in proton ATPases. Data obtained using SIFT and Polyphen-2 bioinformatics software provided additional support for the pathogenic effect of this mutation. Furthermore, we did not find this mutation in 110 Tunisian healthy subjects.

Remarkably, the c.1155dupC mutation was observed in homozygosis in 12 patients from 9 families (cases 2.1, 3.1, 3.2, 4.1, 6.1, 7.1, 7.2, 8.1, 10.1, 10.2, 12.1 and 13.1). In 11 of these 12 patients, SNHL was evident since childhood, but in one of them with normal hearing capability at diagnosis (case 7.2, diagnosed by molecular familiar screening), hypoacusia appeared later. This frameshift mutation results in the generation of a premature stop codon after a short segment from the generated novel triplet. Parents of the siblings 10.1, 10.2, 12.1 and 13.1 were heterozygous, thus carriers for that mutation. The other parents’ samples were not available for genetic testing.

Another mutation in intron 2, affecting the splice acceptor site (c.175-1G > C; p.?), was identified in three probands (1.1, 9.1 and 11.1) from three different families. Mother of 9.1 (father’s sample was not available) and both parents of 11.1 were found to be heterozygous for this mutation. This mutation predicts the loss of the acceptor splice site [11].

In addition, we found a novel heterozygous polymorphic change in intron 5 (c.368-27 C > T) in four patients from four unrelated families (cases 1.1, 8.1, 11.1 and 14.1).

### Mutations in the *ATP6V0A4* gene

Screening for *ATP6V0A4* mutations in 7 probands (patients 14.1, 15.1, 16.1, 17.1, 18.1, 19.1 and 20.1) showed the presence of four different mutations in these 7 families (Table 2): two missense (c.1739 T > C; p.Met580Thr in exon 17 and c.2035G > T; p.Asp679Tyr in exon 19), one nonsense (c.16C > T; p.Arg6* in exon 3) and one frameshift (c.1221delG; p.Met408Cysfs*10 in exon 13). The latter is a single-base deletion leading to a premature stop codon, predicting the consequent synthesis of a truncated protein, lacking all the trans-membrane domains. This mutation found in our series (case 14.1) has previously been published as a case report [23]. Patient’s mother was found to be heterozygous, thus carrier for the mutation (father’s sample was not available).

The missense c.2035G > T mutation was found in two siblings (cases 16.1 and 16.2) in heterozygosis (their father was also heterozygous for this mutation). It leads to the change of aspartic acid in position 679 by tyrosine [11]. In this family, we were not able to find the second mutation responsible for recessive dRTA.

The second missense c.1739 T > C mutation was found in one proband (case 15.1) in heterozygosis. This mutation was described to be located in a predicted transmembrane segment [8]. The substitution of methionine in position 580 by threonine is likely to disrupt secondary structure, function or both.

The nonsense c.16C > T mutation was identified in four families (cases 15.1, 17.1, 18.1 and 20.1). It causes the substitution of Arg6 by a premature stop codon. Prediction analysis showed that this nonsense mutation should lead to an abnormally short protein of only 6 amino acids (p.Arg6*). Further, mother of 17.1, father of 18.1 and both parents of 20.1 were found to be heterozygous for the mutation.

In addition, we found three polymorphic changes in both heterozygosis or homozygosis: (c.5 T > C; p.Val2Ala) in exon 3 in 7 patients (14.1, 15.1, 16.2, 17.1, 18.1, 19.1 and 20.1), c.1662C > T; p.Phe554Phe in exon 16 in three patients (16.1, 16.2 and 19.1) and c.1812 T > C; p.His604His in exon 17 in 8 patients (14.1, 15.1, 16.1, 16.2, 17.1, 18.1, 19.1 and 20.1).

## Discussion

This current study is, to our knowledge, the first large Tunisian dRTA cohort that has been genetically studied so far. As a whole, we identified three different *ATP6V1B1* gene mutations (in 13 families), and four *ATP6V0A4* mutations (in 7 families) in 25 Tunisian children. Remarkably, one novel mutations was observed, c.1102G > A; p.Glu368Lys in the *ATP6V1B1* gene. On the other hand, most studied patients were deaf since early childhood and, remarkably, 75% of them (12/16) had the *ATP6V1B1* gene c.1155dupC mutation [7] in homozygosis, a finding that may improve genetic diagnosis in Tunisia and close populations.

Despite primary dRTA is a rare disease of unknown prevalence, we found a surprising large number of affected Tunisian patients, a country with an estimated population of 10,4 million inhabitants and a mean endogamy rate of 26% [24]. This can be explained, almost in part, by the high rate of consanguinity found in our study cohort, up to 80%. Patients’ clinical presentation was consistent with the prototypic picture of primary dRTA, and every patient showed inability to acidify urine in presence of non-gap metabolic acidosis [1,3,19]. In our series, *ATP6V1B1* gene causative mutations were 2 time-fold more frequent than *ATP6V0A4* gene mutations: 13 vs. 7. This finding may be related not only to ethnicity or the studied population’s characteristics, but also to an easier recognizable pattern of severe acidosis associated with early onset SNHL, leading straightforward to the disease clinical diagnosis [1,7].

Similar presentation with early onset of non-gap metabolic acidosis, hypokalemia and nephrocalcinosis was observed in almost all patients despite carrying different mutations. No phenotype-genotype correlation in dRTA with SNHL was detected in this cohort, as previously described [10]. On the other hand, this study confirms the association between *ATP6V1B1* mutations and dRTA with early onset SNHL since childhood, and *ATP6V0A4* mutations with dRTA but normal hearing (at least until young adulthood), as reported in the original disease genetic descriptions [7,8,18,25,26], but conflicts with some other reports [9,11], which described *ATP6V0A4* gene mutations to be more frequent than *ATP6V1B1* ones.

However, with more recent clinical follow up data, we have become aware that hearing impairment may represent a spectrum in dRTA, and hearing loss may also develop at an older age in a number of those affected patients with *ATP6V0A4* mutations, but likely less severe than in those with *ATP6V1B1* mutations [9]. Indeed, two siblings (cases 16.1 and 16.2) carrying *ATP6V0A4* mutations had dRTA with SNHL since childhood, as described [11]. Reversely, patients with *ATP6V1B1* mutations may not present hearing loss [11]. One of our patients with the *ATP6V1B1* gene c.1155dupC mutation (case 7.2) developed hypoacusia after diagnosis. In general, these findings raise the question regarding potential mechanisms involved in the effect of different subunit mutations of the same proton pump over hearing ability [9].

Importantly, one of the most interesting findings of this study was the detection of *ATP6V1B1* c.1155dupC mutation in homozygosis in 12 patients from 9 families. In all probands, SNHL was evident since childhood, as classically described [7]. This mutation is present in 12 out of 16 (75%) patients with SNHL. This c.1155dupC mutation had previously been described in 12 families, of which 7 are from North Africa [7,9,19]. Therefore, if we consider these studies, performed in North African patients with dRTA , it turns out that approximately 55% of patients suffering from dRTA with early onset SNHL (before 4 years of age) carry this mutation.

On the basis of similar patients’ geographic origin, it could be hypothesized that there might be a founder effect for the frameshift c.1155dupC mutation (Table 3). Nevertheless, a previous study [11] performed in six families with the recurrent p.Ile386fs mutation found they presented with different haplotypes at the *ATP6V1B1* locus, concluding that a founder effect was not the cause of the high prevalence of this mutation in their population. Unfortunately, we could not study *ATP6V1B1* haplotypes in our cohort due to the lack of available relatives, but we plan to test this issue in the future. It is also possible that Ile386 appears as a potential “mutation hotspot” due to a string of seven cytosine residues at the surrounding positions, suggesting a DNA polymerase slippage during replication.

**Table 3:** **Geographic distribution of previously reported dRTA populations carrying the *ATP6V1B1 *** **and *ATP6V0A4 *** **gene mutations described in this study**

**Gene**	**Mutation**	**Origin**	**Number of families**	**Reference**
***ATP6V1B1***	c.1155dupC	Sweden	1	[7]
		Spain	2	
		Saudi Arabia	1	[9]
		Sicily	1	
		Morocco	1	
		Tunisia	1	[11]
		Algeria	5	
		Tunisia	9	Current study
	c.175-1G > C	Tunisia	1	[11]
		Algeria	2	
		Tunisia	3	Current study
	c.1102G > A	Tunisia	1	Current study
***ATP6V0A4***	c.16C > T	Pakistan	1	[11]
		Tunisia	4	Current study
	c.1739 T > C	Turkey	1	[8]
		Tunisia	1	Current study
	c.2035G > T	Pakistan	1	[11]
		Tunisia	1	Current study
	c.1221delG	Tunisia	1	Current study and [23]

Anyhow, this finding may facilitate and help for rapid genetic diagnosis of dRTA in North African patients; and therefore, we propose that in patients with dRTA and SNHL from Northern Africa countries, c.1155dupC mutation in the *ATP6V1B1* gene should be screened first and, then, analysis should continue with additional mutation screening only in those affected subjects in whom c.1155dupC is not found.

Regarding the origin of the other mutations, the splice-site (c.175-1G > C; p.?) defect was described for the first time in three families from Algeria and Tunisia [11]. The nonsense c.16C > T mutation was previously described in a Pakistani family [11]. The two missense c.2035G > T and c.1739 T > C mutations were previously described in one Pakistani [11] and one Turkish family [8], respectively (Table 3).

On the other hand, the newly identified mutation (c.1102G > A; p.Glu368Lys) may provide in the future some insights into A1 subunit structure and function, which are presently unknown in mammals.

The diagnostic strategy based on the presence of SNHL was successfully used in the current study to determine which gene to test first. Vargas-Poussou *et al* performed linkage studies to identify the disease locus in consanguineous families, and then used the age of onset of SNHL to determine which gene to test first in the non-consanguineous families [11]. Comparing with the algorithm followed by these authors, our strategy resulted in a higher mutation detection global rate: 95% versus 79.5%.

Nevertheless, our mutation screening partially failed in three patients (two of them are siblings). We only identified two already described heterozygous mutations (c.2035G > T and c.1739 T > C) in the *ATP6V0A4* gene. We were unable to find the second mutation responsible for the recessive inheritance of the disease, as described by other authors [11], even after screening the *SLC4A1* gene [12,27,28]. We admit that *SLC4A1* gene was a poor candidate for screening as our patients presented severe metabolic acidosis early in life, but we wanted to exclude the possibility of presenting any mutation in the *SLC4A1* gene, as compound heterozygosity has been described [14,29]. Anyway, we cannot rule out the possibility of a potential undetected deletion involving one or more exons of the analyzed genes [30]. Other authors have hypothesized about the possibility of mutations occurring in an unknown regulatory element of either gene, like promoters [31] or that some intronic variants, especially those affecting the normal splicing sites, may also be involved in the pathogenesis of dRTA [11]. More plausible, there might be other genes involved in tubular acidification ability not yet discovered. Haplotype linkage studies should be performed in those affected patients in whom the second mutation is missed and we plan to continue with that project in the future.

## Conclusion

In this report, the genetic analysis of a Tunisian cohort of dRTA patients as well as a new diagnostic strategy to facilitate the genetic testing in North Africans with dRTA and SNHL is presented. The putative dRTA mutation was found in 90% of the index cases (18/20, the other two being heterozygous), thus genetically confirming the clinical diagnosis.

Our findings confirm the association of *ATP6V1B1* mutations and early onset severe SNHL since childhood, and identify delayed hearing loss as a feature associated with *ATP6V0A4* mutations in some patients as well. Based on our results, we propose that in patients of Northern Africa origin with dRTA and SNHL, c.1155dupC mutation in the *ATP6V1B1* gene should be screened first. Further studies with more patients would be needed to confirm this result.

## Competing interests

The authors declare that they have no competing interests.

## Authors’ contributions

DEH made acquisition of the data (the samples), coordinated the data collection, participated in the conception and design of the study, carried out of the genetic studies, interpreted data and wrote the manuscript. GPN participated in the conception and design of the study, carried out of the genetic studies, analysis data, revised critically and made major contribution to the manuscript. SC, SH, AM, MT, NL, WBR, NG, FEA, SM carried out substantial acquisition of the clinical data. JBC, LC, AHK, GA conceived the study, analyzed and interpreted the data and introduce major corrections to the manuscript. All authors revised the manuscript and gave their final approval.

## Pre-publication history

The pre-publication history for this paper can be accessed here:

http://www.biomedcentral.com/1471-2350/14/119/prepub
